# The Dental Society of the State of New York

**Published:** 1887-06

**Authors:** 


### THE DENTAL SOCIETY OF THE STATE OF NEW YORK.

REPORTED FOR THE INDEPENDENT PRACTITIONER.

This society held its nineteenth annual meeting in the Common Council Chamber at Albany, May 11th and 12th.

The greater part of the morning session of the first day was occupied with routine work. The President delivered his annual address, which was received with applause. The Secretary and Treasurer made their annual reports, showing the society to be in a prosperous condition, both as to membership and finances. The Board of Censors made a report through the Secretary, Dr. Frank French, stating that fourteen candidates had presented themselves for examination, of whom five had been found competent, and were recommended for the degree of Master of Dental Surgery (M.D.S.)

The reports were ordered to take the usual course, and it was voted that the recommendations be adopted as read.

Other reports followed, but there was nothing of special importance until those of the District Societies were read. The reports of the Fifth, Sixth, Seventh and Eighth contained resolutions advising the abolition of the degree of Master of Dental Surgery, and recommending that hereafter no degrees be conferred by the State Society. Upon motion of Dr. Line the resolutions were referred to the Board of Censors, to report during the meeting. The report of the Eighth District Society also contained a resolution protesting against the granting of the Whitney memorial prize to any but a permanent or delegate member. Referred to the Committee on By-Laws.

It was moved and carried that the resolution of the Seventh District Society in regard to changes in the code, when two sections seemed to conflict with each other, be referred to the Committee on Dental Law.

The President called attention to the fact that some visiting dentists were present, and extended to them the privilege of the floor.

Dr. E. Parmly Brown read the report of the Committee on Dental Practice. The report was received and filed, and discussed generally.

Dr. Wells asked Dr. Brown for information about the use of cocaine with arsenic.

*Dr. E. P. Brown*—I have been in the habit of making my own arsenical paste for years. I found that with the use of morphia no uniform results were obtained; there would be cases of extreme pain after application. Then I used it in combination with cocaine; they mix very well, and I have not had the severe after-pains that I had with arsenic and morphia alone.

*Dr. Wells*—I am glad to hear this statement, because it shows the therapeutical effects of the agents. It seems to me Dr. Brown's experience shows clearly that cocaine, which is not as good as morphine in some instances, is better in others. I doubt that the arsenic neutralizes the effect of morphia.

*Dr. Brockway*—When I hear these objections made to morphine and cocaine, I think the trouble must be in the agents. I have for many years practiced the taking out of pulps after a simple application of arsenic and creosote, and the operation has been painless. I believe the nerve paste sold in the shops is simply arsenic mixed with creosote.

*Dr. Dwinelle*—I rise to endorse what has been said by Dr. Brockway. I have been in the habit of using a paste of arsenic, creosote and morphine, for the last forty years; I am a little bashful about saying how many more than forty. I have never failed to destroy the pulps without pain; I might almost say I never had a painful result. The trouble may be in the preparation used, but it has been my good fortune during that period to succeed in devitalizing nerves painlessly by that application. Arsenic, as we all know, is a poisonous caustic, and the other two ingredients modify the effect. Morphine is a sedative, and it qualifies the caustive properties of the arsenic. Creosote is also a sedative, and these in combination effect a result which is perfectly satisfactory.

*Dr. Campbell*—I would like to enquire what is the preparation of morphine that you use?

*Dr. Dwinelle*—The sulphate. I presume the acetate would be as effective.

*Dr. Rhein*—In the use of cocaine during pulp devitalization, a clear opening should be made to the pulp, and the finest needle of a hypodermic syringe inserted, and the cocaine forced through it. I have been successful in obtunding a pulp in a few moments. I have never had any failure in using cocaine when I have been able to introduce the point of a hypodermic syringe. I have for that purpose some points much finer than are usually made, with suitable curves to reach the places desired.

*Dr. Tripp*—Dr. Brown says that many preparations of cocaine are worthless; but there is a preparation which renders the excavation of sensitive teeth absolutely painless. It is the alkaloid dissolved in ether.

(TO BE CONTINUED).

